# Analyzing gene expression data for pediatric and adult cancer diagnosis using logic learning machine and standard supervised methods

**DOI:** 10.1186/s12859-019-2953-8

**Published:** 2019-11-22

**Authors:** Damiano Verda, Stefano Parodi, Enrico Ferrari, Marco Muselli

**Affiliations:** 1 Rulex Inc., Newton, MA USA; 2 Epidemiology and Biostatistics Unit, IRCCS Istituto Giannina Gaslini, Genoa, Italy; 3 Institute of Electronics, Computer and Telecommunication Engineering National Research Council of Italy, Via De Marini, 6, 16149 Genoa, Italy

**Keywords:** Logic learning machine, Neural network, Support vector machine, Decision tree, K-nearest neighbor classifier, Gene expression, Microarrays, Cancer, Diagnosis, Prognosis

## Abstract

**Background:**

Logic Learning Machine (LLM) is an innovative method of supervised analysis capable of constructing models based on simple and intelligible rules.

In this investigation the performance of LLM in classifying patients with cancer was evaluated using a set of eight publicly available gene expression databases for cancer diagnosis.

LLM accuracy was assessed by summary ROC curve (*sROC*) analysis and estimated by the area under an *sROC* curve (*sAUC*). Its performance was compared in cross validation with that of standard supervised methods, namely: decision tree, artificial neural network, support vector machine (SVM) and *k*-nearest neighbor classifier.

**Results:**

LLM showed an excellent accuracy (*sAUC* = 0.99, 95%*CI*: 0.98–1.0) and outperformed any other method except SVM.

**Conclusions:**

LLM is a new powerful tool for the analysis of gene expression data for cancer diagnosis. Simple rules generated by LLM could contribute to a better understanding of cancer biology, potentially addressing therapeutic approaches.

## Background

Logic Learning Machine (LLM) is an innovative method of supervised data mining based on an efficient implementation of the Switching Neural Network model. In recent years LLM has been applied to a variety of biomedical settings [1–6]. The advantage of LLM with respect to most traditional methods of supervised data analysis is the capability of identifying simple intelligible rules with potential diagnostic and prognostic applications. In particular, LLM was applied to extract few highly discriminant rules from a signature of 62 genes related to hypoxic condition for the prognosis of neuroblastoma, a highly fatal childhood cancer [2]. In such analysis LLM outperformed standard methods of machine learning, including: Decision Tree (DT), Artificial Neural Network (ANN) and *k*-Nearest Neighbor classifier (kNN). Furthermore, the capability of LLM to exploit the complex correlation structure of highly dimensional gene expression data for feature selection tasks and to combine information from clinical features and gene expression for classification purposes was reported in the analysis of both simulated and real data sets [1, 6]. These results indicate that LLM could be a new powerful and flexible tool for the analysis of gene expression data in Oncology setting. However, its accuracy as classifier when applied to a set of gene expression databases for cancer diagnosis remains to be assessed.

The present study is aimed at evaluating the performance of LLM through an extensive analysis of microarray gene expression data of cancer patients in diagnostic settings. A comparison with standard methods of supervised analysis is also provided.

## Results

### Data set identification

Fifty-two datasets were retrieved from the GEO web site. After a careful examination of their content and the related documentation, 44 were excluded because they did not fully comply with the selection criteria, thus leaving eight data sets available for the analyses. The complete list of excluded datasets and the reason for their exclusion are shown in Table 1. Briefly, nine data sets were excluded due to non-independent sampling (five based on time series analysis and four including matched tissues), 16 for insufficient sample size, one due to the lack of class separation, and one because data were collected from a cancer transplantation in mice. Finally, 17 data sets included samples from only one class of malignancy, thus leaving eight data sets with at least two classes suitable for diagnostic comparisons (7 adult cancers and 1 childhood malignancy).

**Table 1:** Microarray data sets excluded from the analyses and reason for their exclusion

GEO dataset accession	Disease	Reason for exclusion
*GDS4562*	Squamous cell carcinoma of the tongue	Non independent sampling: repeated measures
*GDS4547*	Clear cell renal carcinoma	Non independent sampling: repeated measures
*GDS4395*	Prostate cancer	Non independent sampling: repeated measures
*GDS4284*	Chronic lymphocytic leukaemia	Non independent sampling: repeated measures
*GDS4088*	Breast cancer	Non independent sampling: repeated measures
*GDS4336*	Pancreatic adenocarcinoma	Non independent sampling: different tissues from the same patient
*GDS4282*	Clear cell renal carcinoma	Non independent sampling: different tissues from the same patient
*GDS4176*	Chronic lymphocytic leukaemia	Non independent sampling: different tissues from the same patient
*GDS3829*	Chronic lymphocytic leukaemia	Non independent sampling: different tissues from the same patient
*GDS4515*	Colorectal adenocarcinoma	Insufficient sample size in at least one class
*GDS4470*	Glioblastoma	Insufficient sample size in at least one class
*GDS4379*	Colorectal cancer	Insufficient sample size in at least one class
*GDS4305*	Acute myeloid leukaemia	Insufficient sample size in at least one class
*GDS4299*	Acute lymphoblastic leukaemia	Insufficient sample size in at least one class
*GDS4297*	Acute lymphoblastic leukaemia	Insufficient sample size in at least one class
*GDS4289*	T-lymphoblastic leukaemia	Insufficient sample size in at least one class
*GDS4210*	Acute myeloid leukaemia	Insufficient sample size in at least one class
*GDS4182*	Acute myeloid leukaemia	Insufficient sample size in at least one class
*GDS4168*	Chronic lymphocytic leukaemia	Insufficient sample size in at least one class
*GDS4167*	Chronic lymphocytic leukaemia	Insufficient sample size in at least one class
*GDS4503*	Breast cancer	Insufficient sample size in at least one class
*GDS3885*	Glioblastoma and glioma	Insufficient sample size in at least one class
*GDS3869*	B-cell lymphoma	Insufficient sample size in at least one class
*GDS3716*	Breast cancer	Insufficient sample size in at least one class
*GDS3897*	Inflammatory bowel disease	No malignant cancer and insufficient sample size
*GDS4813*	Malignant melanoma	No available classes to compare
*GDS4381*	Colorectal cancer	Non-human tissue (transplantation on mice)
*GDS4761*	Breast cancer	No available classes for cancer diagnosis
*GDS4718*	Colorectal cancer	No available classes for cancer diagnosis
*GDS4516*	Colorectal cancer	No available classes for cancer diagnosis
*GDS4589*	Stage I endometrial cancer	No available classes for cancer diagnosis
*GDS4513*	Colon cancer	No available classes for cancer diagnosis
*GDS4456*	Bladder cancer	No available classes for cancer diagnosis
*GDS4393*	Colorectal cancer	No available classes for cancer diagnosis
*GDS4278*	Acute myeloid leukaemia	No available classes for cancer diagnosis
*GDS4222*	Hodgkin’s lymphoma	No available classes for cancer diagnosis
*GDS4206*	Acute lymphoid leukaemia	No available classes for cancer diagnosis
*GDS4198*	Gastric adenocarcinoma	No available classes for cancer diagnosis
*GDS4181*	Acute myeloid leukaemia	No available classes for cancer diagnosis
*GDS4109*	Prostate cancer	No available classes for cancer diagnosis
*GDS4057*	Breast cancer	No available classes for cancer diagnosis
*GDS4056*	Breast cancer	No available classes for cancer diagnosis
*GDS3837*	Non-small cell lung cancer	No available classes for cancer diagnosis
*GDS3795*	Myelodisplastic syndrome	No available classes for cancer diagnosis

Table 2 shows the list of the eight databases included in the analyses, ordered by their appearance in the GEO data bank. Three data sets (*GDS4968*, *GDS4296* and *GDS3952*) included multiple-class comparisons (three classes for the first, five for the second and four for the last one), while the remaining five databases (*GDS4887*, *GDS4794*, *GDS4762*, *GDS4471*, and *GDS3945*) included two classes.

**Table 2:** Microarray data sets included in the analyses and the related classes at comparison

GEO dataset accession	*N*	Disease	Classes at comparison
*GDS4968*	99	Multiple myeloma	Monoclonal gammopathy (*n* = 20) vs. multiple myeloma (*n* = 41) vs. smoldering multiple myeloma (*n* = 33)
*GDS4887*	40	Hepatocellular carcinoma	Hepatocellular carcinoma (*n* = 20) vs. non-tumor (*n* = 20)
*GDS4794*	65	Small cell lung cancer	Normal cells (*n* = 42) vs. small cell lung cancer (*n* = 23)
*GDS4762*	80	Breast cancer	Cancer cells (*n* = 37) vs. fibroblasts (*n* = 43)
*GDS4471*	76	Medulloblastoma	Classic medulloblastoma (*n* = 51) vs. other types (*n* = 25)
*GDS4296*	174	Many different malignancies	Renal cancer (*n* = 23) vs. colon cancer (*n* = 21) vs. melanoma (*n* = 26) vs. non-small cell lung cancer (*n* = 29) vs. ovarian cancer (*n* = 21)
*GDS3952*	162	Breast cancer	Benign (*n* = 37) vs. ectopic (*n* = 22) vs. healthy controls (*n* = 31) vs. malignant (*n* = 51)
*GDS3945*	42	Renal clear cell carcinoma	T3 thyronine (*n* = 21) vs. untreated controls (*n* = 21)

*N* number of samples

In more details, the *GDS4968* data set included 99 samples and 33,297 gene features of pre-malignant (monoclonal gammopathy) and malignant (multiple myeloma) bone marrow plasma cells [7]. Three-class comparison was made between a group of gammopathy of undetermined significance (*n* = 20), multiple myeloma (*n* = 41) and smoldering (i.e., asymptomatic) multiple myeloma (*n* = 33); data related to five healthy controls were excluded from the analyses due to the insufficient sample size.

*GDS4887* included 40 samples and 54,675 features of hepatocellular carcinoma diagnosed in patients with chronic hepatitis C [8]. Expression profile of 20 carcinoma tumor was compared to that of 20 non tumor tissues.

*GDS4794* included 23 samples of small cell lung cancer and 42 normal tissues [9]. Gene expression features were 54,675. *GDS4762* included 37 breast cancer cells and 43 fibroblasts [10], and gene expression was measured in 33,297 features.

*GDS4471* included 76 samples of medulloblastoma (a malignant childhood cancer of the brain) from patients aged 3 to 16 years [11]. The analysis was performed comparing the expression of 54,675 features in 51 samples of classic medulloblastoma (MB_CL) with that of a pool of 25 other types (namely: 6 desmoplastic nodular, 17 anaplastic and 2 medullomyoblastoma).

*GDS4296* included 54,675 features and 174 samples from 9 different cancer tissues [12]. Among them the following malignancies were included in the analysis: 23 renal cancers, 21 colon cancers, 26 malignant melanomas, 26 non-small cell lung cancers and 21 ovarian cancers, whereas other tumor types were excluded for insufficient sample size (namely: 15 breast, 18 central nervous system and 6 prostate cancers, and 18 leukemia cases).

*GDS3952* included 162 samples from breast cancer patients and a variety of other malignancies and normal tissues [13]. Comparison between the expression of 54,675 features in the following disease groups was performed: benign breast cancer (*n* = 37), ectopic cancers (*n* = 22), healthy controls (*n* = 31) and malignant breast cancer (*n* = 51); other smaller groups were not considered in the present analysis.

Finally, *GDS3945* included 33,297 features in 42 samples of renal clear cell carcinoma [14]. Comparison was made between 21 samples of renal clear carcinoma cells stimulated by T3 thyronine and 21 samples from healthy controls. Data also included information about different genotypes related to thyronine receptor variants, but data were too sparse to allow for further analyses.

### Comparison of learning machine methods

Table 3 shows the results of the analysis of gene expression profiles in the eight selected data sets by LLM and by the four competing methods. In general, performance was very high for each algorithm in each comparison except for ANN in the *GDS3945* data set. In two cases, including *GDS4794* and *GDS4762*, all machine learning methods performed equally well with the maximum attainable accuracy (100%), except for ANN in the later data set. With regard to the other six comparisons, performance of SVM (measured by the Cohen’s kappa index) was the highest in each analysis, except for *GDS3952*, where kNN showed the best accuracy, and *GDS3945*, where LLM performed better. However, in the *GDS4887* data set ANN and kNN had the same performance of SVM, and in the analysis of *GDS4296* also kNN performed equally high.

**Table 3:** Analysis of gene expression profiles in eight selected data sets of for cancer diagnosis. Comparison between five methods of supervised data mining in cross-validation

Method	Sens. %	Spec. %	Youden Index %	Empirical Accuracy %	Cohen’s Kappa %	*p*
*GDS4968*						
LLM	98.1	90.2	88.4	94.7	91.7	*< 0.001*
DT	96.2	95.1	93.1	95.7	93.3	*< 0.001*
ANN	94.3	95.1	89.4	93.6	90.0	*< 0.001*
SVM	98.1	97.5	95.7	97.9	96.7	*< 0.001*
kNN	98.1	97.6	95.7	96.8	95.1	*< 0.001*
*GDS4887*						
LLM	100	95.0	95.0	97.5	95.0	*< 0.001*
DT	100	95.0	95.0	97.5	95.0	*< 0.001*
ANN	100	100	100	100	100	*< 0.001*
SVM	100	100	100	100	100	*< 0.001*
kNN	100	100	100	100	100	*< 0.001*
*GDS4794*						
LLM	100	100	100	100	100	*< 0.001*
DT	100	100	100	100	100	*< 0.001*
ANN	100	100	100	100	100	*< 0.001*
SVM	100	100	100	100	100	*< 0.001*
kNN	100	100	100	100	100	*< 0.001*
*GDS4762*						
LLM	100	100	100	100	100	*< 0.001*
DT	100	100	100	100	100	*< 0.001*
ANN	97.3	100	97.3	98.8	97.5	*< 0.001*
SVM	100	100	100	100	100	*< 0.001*
kNN	100	100	100	100	100	*< 0.001*
*GDS4471*						
LLM	99.0	96.0	95.0	97.4	94.0	*< 0.001*
DT	88.2	76.0	64.2	84.2	64.2	*< 0.001*
ANN	82.4	88.0	70.4	84.2	66.3	*< 0.001*
SVM	98.0	96.0	94.0	97.4	97.4	*< 0.001*
kNN	94.2	96.0	90.1	94.7	88.3	*< 0.001*
*GDS4296*						
LLM	97.8	96.2	94.0	96.6	95.7	*< 0.001*
DT	75.8	100	75.8	63.3	53.1	*< 0.001*
ANN	98.9	96.2	95.1	93.2	91.4	*< 0.001*
SVM	100	100	100	100	100	*< 0.001*
kNN	100	100	100	100	100	*< 0.001*
*GDS3952*						
LLM	96.4	90.3	87.6	92.2	89.4	*< 0.001*
DT	94.5	100	94.5	70.2	57.7	*< 0.001*
ANN	97.3	90.3	87.6	76.6	67.2	*< 0.001*
SVM	100	100	100	95.7	94.2	*< 0.001*
kNN	100	100	100	97.2	96.1	*< 0.001*
*GDS3945*						
LLM	100	100	100	100	100	*< 0.001*
DT	90.5	100	90.5	95.2	90.5	*< 0.001*
ANN	14.3	81.0	−4.8	46.6	−4.8	*0.661*
SVM	95.2	100	100	97.6	95.2	*< 0.001*
kNN	85.7	95.2	81.0	90.5	81.0	*< 0.001*

**Fig. 1:**
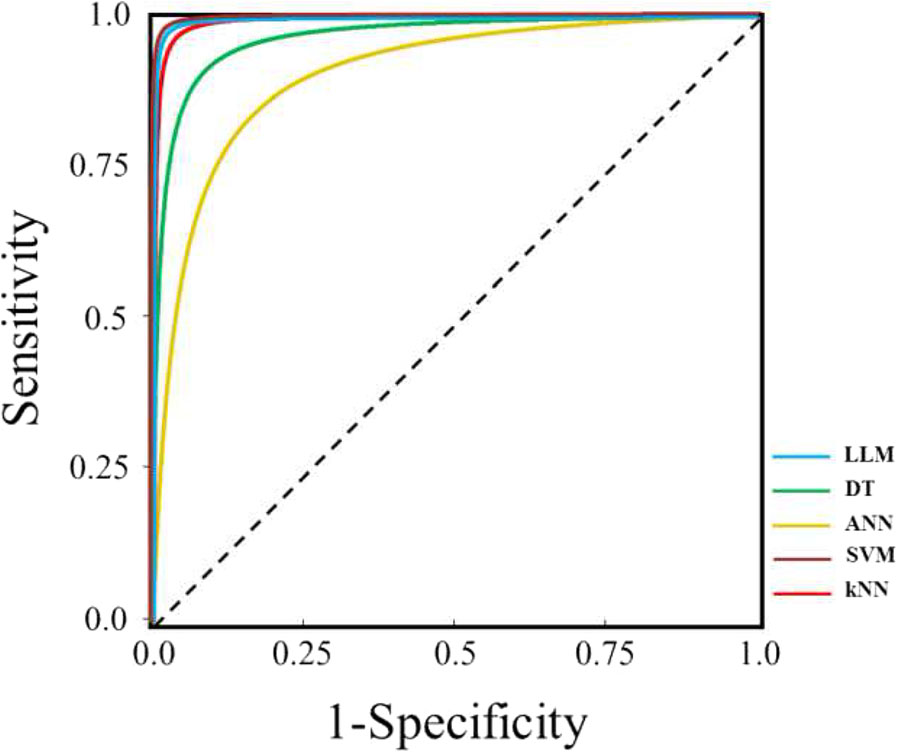
Summary ROC curves for the eight diagnostic comparisons

Figure 1 shows the corresponding summary ROC curves, obtained under a proper model assumption, while the corresponding areas under the curves (sAUC) and the related diagnostic Odds Ratios are reported in Table 4. SVM and LLM outperformed any other method (*sAUC* = 0.996 and 0.995, respectively). kNN and DT also showed an excellent accuracy (sAUC = 0.991 and 0.964), respectively, while ANN had a quite lower accuracy (sAUC = 0.904).

**Table 4:** Results of summary ROC analysis

Method	*sAUC*	*95%CI*	*sOR*	*95%CI*
Diagnostic studies				
*LLM*	0.995	*0.987–0.998*	1546	*375–6363*
*DT*	0.964	*0.933–0.982*	104	*44–251*
*ANN*	0.904	*0.830–0.949*	26	*10–65*
*SVM*	0.996	*0.988–0.999*	1736	*401–7512*
*kNN*	0.991	*0.976–0.997*	635	*177–2279*

*sAUC* summary Area Under the ROC Curve, *95%CI* 95% confidence interval, *sOR* summary Odds Ratio

### Classification rules generated by the logic learning machine

Table 5 illustrates the classification rules extracted by the LLM from each data set. In four analyses, corresponding to *GDS4887*, *GDS4794*, *GDS4762* and *GDS3945* data sets, the LLM classifier included very few simple rules, based on only one condition and two features (one for each class at comparison). Conversely (and not surprisingly), more complex rules were needed to classify samples from the three datasets that included multiple classes (*GDS4968*, *GDS4296*, and *GDS3952*). Three conditions based on a small set of features were sufficient to reach a satisfactory classification accuracy, except for the analysis of the *GDS3952* data set (the corresponding very complex rules had a low covering and were not included in Table 5). Furthermore, the second rule for the classification of monoclonal gammopathies in the *GDS4968* data set included a feature generated by artificial DNA used as an internal control (named “Control_3389”), thus indicating that the classification was partly based on an artifact; covering of the corresponding rule was rather high, i.e.*,* 60%. In the absence of available reliable measures of the concentration of the corresponding mRNA (e.g., by quantitative PCR) it is impossible to establish if such an artifact can be attributable either to a defective extraction of the LLM algorithm or to noising data related to the intrinsic variability of the microarray technique. Finally, in the *GDS4471* data set four classification rules, including a maximum of two conditions each and 6 different features, were identified to separate 41 samples of the classic form of a childhood brain cancer (medulloblastoma) for a pool of 25 heterogeneous samples of rare variants of the same malignancy.

**Table 5:** Classification rules identified by Logic Learning Machine applied to gene expression profiles in eight selected data sets for cancer diagnosis

Output	Condition 1	Condition 2	Condition 3	*Covering*
*GDS4968*				
Monoc. Gamm.	SNHG3_1 ≤ 9.28	SNORA14B ≤ 4.30	–	95.0%
Monoc. Gamm.	Control_3389 ≤ 8.20	–	–	60.0%
MM	THOP1 > 6.23	TARP_5 ≤ 6.71	–	85.4%
MM	C22orf23 ≤ 5.20	FLJ20712 ≤ 3.14	–	26.8%
Smold. MM	DNAJC7 > 8.13	IGK_2 ≤ 10.4561	DEK > 6.50	97.0%
Smold. MM	HNRNPA1 > 6.44	–	–	51.5%
*GDS4887*				
HC	AQP7 ≤ 8.46	–	–	100%
Non tumor	CLPX_1 > 11.4116	–	–	100%
*GDS4794*				
Normal cells	DSCC1_1 ≤ 110.1	–	–	100%
SCLC	CBX3_1 > 2232.75	–	–	100%
*GDS4762*				
Breast cancer	FMN2 < = 116.32	–	–	100%
Fibroblast	SHC4 > 52.20	–	–	100%
*GDS4471*				
Classic MB	EFHD2_1 > 3.87	LOC100132891 ≤ 4.37	–	88.3%
Classic MB	TCL1A > 4.66	–	–	31.4%
Other MB	LOC100132891 > 4.18	5.47 < ZMYM5_3 ≤ 6.17		76.0%
Other MB	CHIAP2 > 3.38	ZNF212 ≤ 6.45	–	40.0%
*GDS4296*				
Colon cancer	KLK6 > 7.71	–	–	100%
Melanoma	EDNRB > 5.72279	–	–	100%
Non-SCLC	5.61 < TMEM51 ≤ 6.55	FAM177A1 > 8.27	LINC00936 > 5.59	100%
Ovarian cancer	TMEM101 ≤ 6.15	–	–	85%
Ovarian cancer	MEIS1_1 > 6.70	–	–	57.1%
Renal cancer	LRRN4 > 4.69	APBB1IP_2 > 7.46	–	100%
*GDS3952*				
Benign disease^a^	2.32 < IGHV7–81 ≤ 3.29	2.87 < BM983749 ≤ 4.06	LIM2 > 4.11	83.8%
Benign disease^a^	LCP2_1 > 9.07	ST8SIA2_1 ≤ 2.215	–	27.0%
Ectopic cancers	ST3GAL1 > 6.55	PWWP2A > 6.18	–	100%
Healthy controls	USMG5 > 11.85	–	–	90.3%
Healthy controls	NUFIP2_1 > 8.81	–	–	41.9%
Breast cancer^a^	MKNK1 ≤ 3.91	227762_at ≤8.3	BF194770 > 2.385	80.4%
Breast cancer	ZNF81 ≤ 2.99	MMAB_1 ≤ 4.095	–	29.4%
Breast cancer	AU143882 > 4.57	–	–	21.6%
*GDS3945*				
Untreated controls	COQ10A < = 125.66	–	–	100%
Renal cancer	COQ10A > 125.66	–	–	100%

*Monoc. Gamm.* Monoclonal Gammopathy, *MM* Multiple Myeloma, *Smold. MM* Smoldering Multple Myeloma, *SCLC* Small Cell Lung Cancer, *HC* Hepatocellular Carcinoma, *MB* Medulloblastoma

^a^Classification algorithm truncated to the first three rules with the highest covering

## Discussion

LLM is an innovative method of supervised analysis that can identify simple and intelligible rules for classification tasks. In previous investigations its accuracy was comparable to that of most common supervised methods based on black-box algorithms and also outperformed DT, which shares with LLM the capability to generate intelligible rules [1–3, 15, 16].

In the present investigation LLM showed an excellent performance in the analysis of dataset for cancer diagnosis. Comparisons included different types of malignancies or different classes of cancer and non-cancer cells. Carcinogenesis is characterized by the disruption of several biochemical paths; then, many to several genes are expected to be strongly differently expressed between different tumor types and, even more so, between neoplastic and non-neoplastic tissues [17]. Contrarily to other rule-based methods, including DT, LLM classification is based on a set of partially overlying rules. Such rules are able to identify small subgroups of interest that tend to escape classification methods based either on univariate analysis or on a divide-and-conquer approach.

An advantage of LLM and DT compared to black-box classifiers is that classification rules can provide useful insights about cancer biology. In some instances, the potential biological role of those genes identified by the simple one-feature LLM rules can be confirmed by evidence from recent studies on independent cohorts of cancer patients. For example, in our study LLM revealed a downregulation of the *Aqp7* gene in a set of 20 hepatocellular carcinoma samples (Table 5, *GDS4887* data set). *Aqp7* encodes for a transmembrane channel protein belonging to the aquagliceroporins family, whose function is the regulation of transcellular movements of water and glycerol in many mammalian tissues [18]. A significative reduction of AQP7 was reported in hepatocellular cancer cells by a recent investigation on 68 patients. The study also found an upregulation of AQP9 and a downregulation of AQP3 (two molecules belonging to the same family of AQP7), indicating that the dysregulation of the aquaporine activity could play a fundamental role in the liver cancer development and progression [19].

In the *GDS4794* data set LLM found an overexpression of *Cbx3* in 23 small cell lung cancer samples. CBX3 is a member of the heterochromatin protein 1 family that plays a fundamental role in multiple cellular functions, including DNA repair, transcriptional regulation, and telomere function [20]. CBX3 has been suspected to be responsible of the silencing of tumor suppressor genes and it was demonstrated to be upregulated in many tumors. Interestingly, in a recent investigation, Chang et al. [21] found a high prevalence of samples positive for the CBX3 in a set of 88 lung cancer patients. Most subjects were affected by lung adenocarcinoma, while samples in the *GDS4794* database belonged to another subtype of lung cancer (namely: the small cell one). Our finding, if confirmed by further independent investigations, could provide some insights to the knowledge of the carcinogenesis of the different subtypes of lung cancer.

Composite rules generated by combining different features are more difficult to interpret, but they could shed some light on the complex interaction at the basis of carcinogenesis and tumor progression. In the *GDS4471* data set, LLM found an overexpression of the *Efhd2* gene that, combined to a downregulation of *Loc100132891*, was able to separate most samples of classic medulloblastoma (88.3%) from the other subtypes of the same tumor. Medulloblastoma is a childhood cancer, whose different subtypes can be associated with different origins and patient survival [11]. Difference in gene expression between different tumor subtypes could help both to provide some insights on the carcinogenesis process and to identify potential targets for new therapeutic approaches. In this example, the overexpression of *Efhd2* (also commonly known as *swiprosin-1*) is not a surprising finding, in that it encodes for a calcium ion binding protein involved in several cellular functions, including neurodegeneration and cancer [22]. Conversely, *Loc100132821* is a long non-coding RNA whose function is, at least to our knowledge, still unknown (*http://www.igenebio.com/gene/hs-loc100132891.html*). In the two last decades many long non-coding transcripts have been found to play a crucial role in carcinogenesis [23], then the possibility that a reduced transcription of *Loc100132821* in cells with a high expression of *swiprosin-1* could be involved in the carcinogenesis of classic medulloblastoma might be a new finding that deserves further investigations.

The present study represents the first analysis comparing LLM with different supervised methods for cancer diagnosis using a set of microarray data including different cancer types. LLM showed an excellent accuracy, but our results should be interpreted at the light of some limitations of this study. First of all, this should be considered as a pilot study comparing classification methods under similar simplified conditions and the proposed method should be applied to larger sets of microarray data, including more difficult clinical context (e.g., evaluation of patients’ prognosis, response to drug treatments, vaccination trials, etc.). In particular, the tuning methods have produced a very high classification performance that, in the absence of an independent validation cohort, should be interpreted as an upper limit of the actual expected classification accuracy.

In spite of the extensive search in the GEO repository, only eight data sets with a sufficiently high dimension have been identified, then the excellent performance found for LLM should be confirmed by further independent studies. Baseline conditions that can influence the LLM performance are still to be elucidated, and further analyses based on large data sets including both real and ad hoc simulated data are needed, in particular to assess the effect of different sample size, unbalanced vs. balanced design, noisy data, and poorly defined gold standards.

Finally, in this study, similarly to some other previous investigations [1, 2, 16], LLM outperformed DT, which shares with our method the capability to extract simple rules for classification purposes. At least at our knowledge, DT is the most largely employed method of machine learning based on simple threshold rules. However, in the last decades some other innovative methods have been proposed and applied for classification purposes in many biomedical fields [24, 25], then in further investigations the accuracy of LLM should be also compared to that of such methods.

## Conclusions

LLM is an innovative method of supervised analysis that has shown a very high accuracy in classifying patients with different oncological conditions.

The LLM classifier is typically based on a small set of simple and intelligible rules that could contribute to a better understanding of carcinogenesis process, hopefully supporting the adoption of new therapeutic approaches.

## Methods

### Data selection

Data were drawn from a set of publicly available databases of gene expression microarrays, stored in the GEO repository bank (http://www.ncbi.nlm.nih.gov/gds/). Selection criteria were: a) inclusion in the GEO data bank from January 2010 to December 2014; b) presence of at least two classes potentially useful for cancer diagnosis, including at least 20 samples each; c) availability of a scientific paper in English language, published on PubMed, and fully describing the experiment and the related study design.

An early selection was made using the following key words in the GEO website: *cancer AND human [Organism] AND 40:10000[Number of Samples] AND 2010/01:2014/12[Publication Date] AND GDS[ETYP] AND “gds PubMed”[Filter]*.

The retrieved databases were carefully investigated to assess their full compliance to the selection criteria. Moreover, studies based on a matched design were excluded, because all the applied methods of analysis rely on the assumption of independent sampling.

### Logic learning machine (LLM)

LLM generates classifiers described by a set of intelligible rules of the type:

$$ \mathbf{if}< premise>\mathbf{then}< consequence> $$

where <premise> is a logical product (AND) of conditions and < consequence> provides a class assignment for the output [5, 6, 15].

**Fig. 2:**
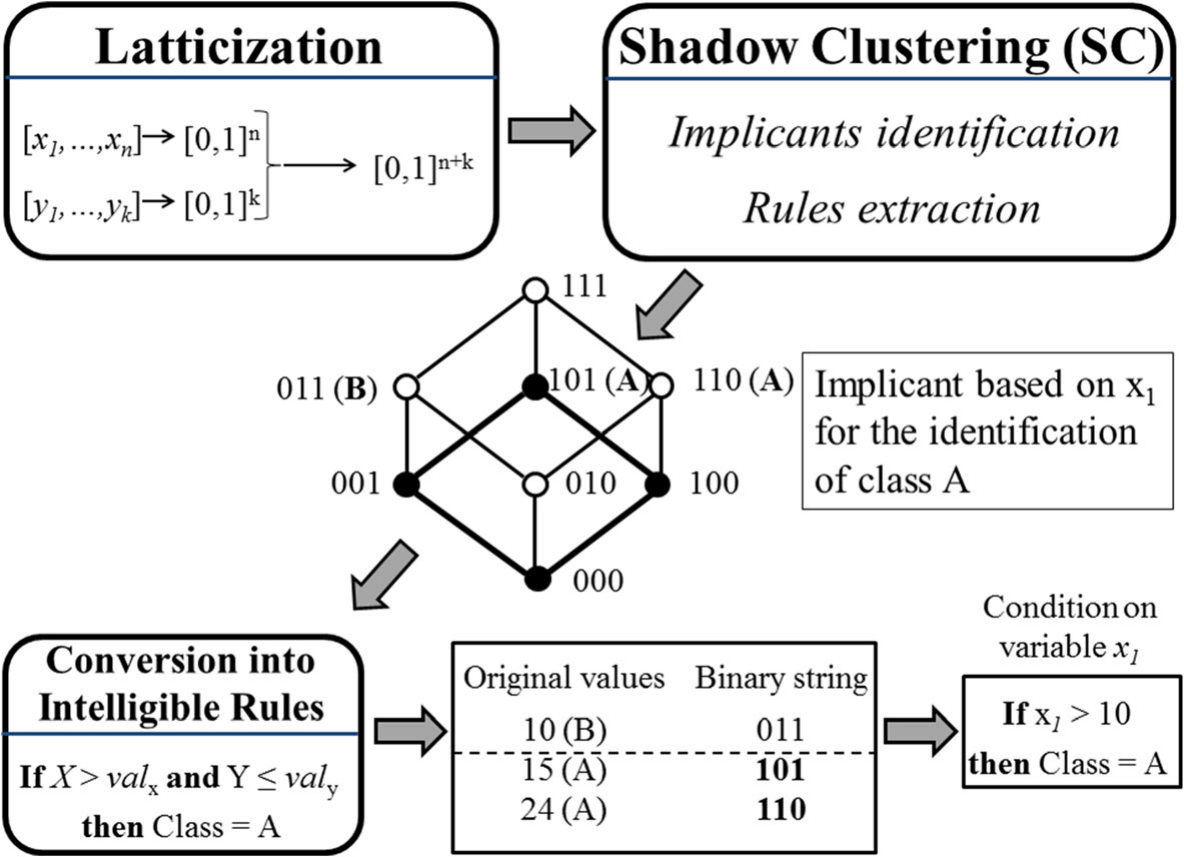
Schematic representation of the Logic Learning Machine algorithm. In the first phase (Latticization) each variable is transformed into a string of binary data, using the inverse only-one code binarization and all strings are eventually concatenated in one unique large string per each subject. In the second phase (Shadow Clustering) a set of binary vectors (the “implicants”) is generated, each of which identifies a cluster in the input space associated with a specific output class. Finally, all the implicants are transformed into simple conditions and combined in a set of intelligible rules

LLM produces rules through a three-step process, namely: latticization (binarization), monotone Boolean function reconstruction and rule generation (Fig. 2). In the first phase (latticization) each variable is transformed into a string of binary data, using the inverse only-one coding [15]; then, resulting coded strings are concatenated in one unique large sequence of bits. In the second phase (monotone Boolean function reconstruction) a set of binary vectors, called implicants, is selected and allows the identification of clusters associated with a specific class. During the third phase all the generated implicants are transformed into as many rules, each one including a collection of simple threshold conditions in its <premise> part. Algorithms for the efficient generations of implicants, starting from any dataset, have been illustrated in detail elsewhere [15].

A set of quality measures has been defined for any rule *r* generated by LLM [2, 15], such as the proportion of correct classifications *C*(*r*), called the covering, or the false positive fraction *E*(*r*). In a binary classification task, depending on the class identified by the rule *r*, *C*(*r*) will correspond to either the sensitivity or the specificity. Let *r’* represent the rule obtained from *r* by removing the condition *c* from its premise part. A simple measure of the relevance *R*(*c*) of that condition is then provided by:

$$ R(c)=\Delta  E(c)C(r) $$

where

$$ \Delta  E(c)=E\left(r^{\prime}\right)-E(r) $$

Finally, a measure of relevance *R*_*v*_(*x*_*j*_) for each variable *x*_*j*_ can be obtained by applying the following equation:

$$ {R}_v\left({x}_j\right)=1-\prod \limits_k\left(1-R\left({c}_{kl}\right)\right) $$

where *k* varies on the indices of rules *r*_*k*_ that includes a condition *c*_*kl*_ on the variable *x*_*j*_.

As a rule of thumb, the inequality *R*_*v*_*(x*_*j*_*)* ≤ 10% is used to identify a predictor *x*_*j*_ providing a marginal contribution to the accuracy of LLM classifiers, while a rule with *C*(*r*)(1 – *E*(*r*)) ≤ 10% often covers subjects with anomalous values (possible outliers).

### Accuracy assessment

#### Measures of quality for a single analysis

Performance of LLM was compared with that of four selected competing methods of supervised learning (DT, ANN, SVM, and kNN) in leave-one-out cross-validation (LOOCV). Standard measures of quality were obtained for each analysis and proper comparison techniques were adopted to evaluate the overall performance of each classification method in the whole set of analysis. A parameter tuning procedure was adopted to enhance the performance of each selected method and only the models with the highest accuracy were retained. For instance, for LLM a set of values for the *E*(*r*) parameter were selected, ranging from 2.5 to 7.5% (step 0.5%). Parameter tuning for the competing methods will be described in the dedicated paragraphs.

Consider a two-class classification problem, where the output can assume two different values identified as positive and negative. Each analysis with any supervised learning method is characterized by four values:

- The number *TP* of positive samples correctly classified by the resulting model,
- The number *FN* of positive samples wrongly classified by the resulting model,
- The number *TN* of negative samples correctly classified by the resulting model,
- The number *FP* of negative samples wrongly classified by the resulting model.

From these four values other quality measures for the analysis can be derived, among which:

- the *sensitivity SE* and the *specificity SP*:

$$ SE=\frac{TP}{TP+ FN}\kern0.5em SP=\frac{TN\ }{TN+ FP} $$

- the *Youden index Y*:

$$ Y= SE+ SP-1 $$

- the *Cohen kappa coefficient K*:

$$ K=\frac{2\left( TP\bullet TN- FP\bullet FN\right)}{\left( TP+ FP\right)\left( TN+ FP\right)+\left( TN+ FN\right)\left( TP+ FN\right)} $$

- the *odds ratio OR*:

1 $$ OR=\frac{SE\bullet SP}{\left(1- SE\right)\left(1- SP\right)} $$

In the present investigation we have log-transformed the *OR* to exploit its asymptotic Normal distribution [26]. An asymptotic estimate of the variance σ^2^ of the logarithm log(*OR*) of the odds ratio is readily obtained through the equation [27]:

$$ {\sigma}^2=\frac{1}{TP}+\frac{1}{FN}+\frac{1}{TN}+\frac{1}{FP} $$

where the continuity correction is adopted if one of the terms at the denominator is null [27].

In the presence of *m* multiple outcomes, the definition of the Cohen kappa coefficient can be generalized as follows [28]:

$$ K=\frac{\sum \limits_{i=1}^m{a}_{ii}-\sum \limits_{i=1}^m{e}_{ii}}{\sum \limits_{j=1}^m\sum \limits_{i=1}^m{a}_{ij}-\sum \limits_{i=1}^m{e}_{ii}} $$

where a_ij_ represents the counting of elements in the i row and j column of the confusion matrix and

$$ {e}_{ij}=\frac{\sum \limits_{i=1}^m{a}_{ij}\sum \limits_{j=1}^m{a}_{ij}}{\sum \limits_{j=1}^m\sum \limits_{i=1}^m{a}_{ij}} $$

represents the corresponding expected counting in the case of a random distribution of the elements inside the cells of the confusion matrix.

The specificity *SP*_*m*_ for *m* multiple outcomes was simply obtained by selecting a category as the reference and computing the proportion of correctly classified samples inside that category.

The corresponding estimate of log(*OR*) and of its related variance σ^2^ were then retrieved by applying the Mantel Haenszel (MH) method [29]. Let *i* = 1 be the index of the reference category in the confusion matrix; the MH estimate of log(*OR*) is obtained as:

$$ \log (OR)\cong \log \left(\frac{\sum \limits_{i=2}^m\frac{a_{11}{a}_{ii}}{a_{11}+{a}_{1i}+{a}_{i1}+{a}_{ii}}}{\sum \limits_{i=2}^m\frac{a_{1i}{a}_{i1}}{a_{11}+{a}_{1i}+{a}_{i1}+{a}_{ii}}}\right) $$

whereas the corresponding asymptotic estimate of the variance σ^2^ is obtained through the following equation [30]:

$$ {\sigma}^2\cong \frac{\sum \limits_{i=2}^m\frac{\left({a}_{11}+{a}_{1i}\right)\left({a}_{i1}+{a}_{ii}\right)\left({a}_{11}+{a}_{i1}\right)\left({a}_{1i}+{a}_{ii}\right)}{{\left({a}_{11}+{a}_{1i}+{a}_{i1}+{a}_{ii}-1\right)\left({a}_{11}+{a}_{1i}+{a}_{i1}+{a}_{ii}\right)}^2}}{\left(\sum \limits_{i=2}^m\frac{a_{1i}{a}_{i1}}{a_{11}+{a}_{1i}+{a}_{i1}+{a}_{ii}}\right)\left(\sum \limits_{i=2}^m\frac{a_{11}{a}_{ii}}{a_{11}+{a}_{1i}+{a}_{i1}+{a}_{ii}}\right)} $$

Finally, the sensitivity *SE* for multiple outcomes is obtained by exploiting the relationship between *OR*, *SE* and *SP* reported in eq. (1):

$$ SE=\frac{OR\left(1- SP\right)}{SP+ OR\left(1- SP\right)} $$

A “natural” reference category for multiple outcomes was adopted, whenever possible, selecting either the group of subjects without any disease, if any, or the class with the (allegedly) less severe illness. Otherwise, in the case of comparison between groups of severely diseased patients (i.e., classes including only malignant tumors) the reference was arbitrarily defined as the class with the highest number of individuals.

#### Common measures of quality across studies: the summary ROC curve

For each dataset, comparison between the considered supervised classification methods was based on the *K* index.

For each classifier a common measure of accuracy across the *N* studies was obtained by employing the method of the summary ROC (*sROC*) curves [31]. In particular, the area *sAUC* under the *sROC* curve was adopted to evaluate the quality of any classification technique. A proper model was considered, which is described by the following equation:

$$ sROC(x)=\frac{x\bullet sOR}{x\bullet sOR+1-x} $$

where *sOR* is the summary odds ratio given by:

$$ sOR=\exp \left(\sum \limits_{i=1}^N\frac{\mathit{\log}\left({OR}_i\right)}{\sigma_i^2}\right) $$

being *OR*_*i*_ the odds ratio of the *i*th study and σ_*i*_^2^ the variance of log(*OR*_*i*_).

An estimate of the standard error for log(*sOR*) can also be obtained as

$$ StdErr\left[\log (sOR)\right]=\sqrt{\frac{1}{\sum \limits_{i=1}^N\raisebox{1ex}{$1$}\!\left/ \!\raisebox{-1ex}{${\sigma}_i^2$}\right.}} $$

whereas, under the log-Normal assumption for the distribution of *sOR*, the related 95% confidence intervals (95%*CI*) of this estimate are obtained as follows:

$$ 95\% CI=\exp \left( sOR\pm 1.96\bullet \sqrt{\frac{1}{\sum \limits_{i=1}^N\raisebox{1ex}{$1$}\!\left/ \!\raisebox{-1ex}{${\sigma}_i^2$}\right.}}\right) $$

The value of *sAUC* represents a summary measure of pure accuracy [31] and is easily obtained from *sOR* through the following equation:

$$ sAUC=\frac{sOR}{sOR-1}-\frac{sOR\bullet \log (sOR)}{{\left( sOR-1\right)}^2} $$

In the present study we have performed an *sROC* analysis for each of the five classification methods thus resulting in five *sROC* curves.

All supervised analyses were carried out by using Rulex, a software suite developed and commercialized by Rulex Inc. (http://www.rulex.ai). Summary ROC analysis was performed by Stata for Windows statistical software (release 12.1, Stata Corporation, College Station, TX).

### Brief description of competing methods of supervised data mining

#### Decision tree (DT)

A DT is a graph where each node is associated with a condition based on an attribute of the input vector ***x*** and each leaf corresponds to an assignment for a specified output class. Moving from a leaf to a root, a simple intelligible rule can be identified [32].

DT is obtained by a “divide-and-conquer” approach that provides disjoint rules. At each iteration, a new node is added to the DT by choosing the condition that subdivides the training set *S* according to a specific measure of goodness. Parameter tuning was performed comparing the performance of three different pruning approaches (namely: pessimistic, no pruning and cost-complexity). Furthermore, the highest impurity by node was let to vary between 0.0 and 0.1 (step 0.01).

#### Artificial neural network (ANN)

ANN is a connectionist model formed by the interconnection of simple units (neurons), arranged in layers. Each neuron computes a weighted sum of the inputs applying a proper activation function, which provides the output value that will be propagated to the following layer. The input vector ***x*** is sent to the first layer. The remaining layer receives input from the previous one and the last layer produces the output class to be assigned to ***x***. Weights for each neuron are estimated by suitable optimization techniques and form the set of parameters for the ANN. The Levenberg-Marquardt version of the back propagation algorithm was applied to train the ANN [32]. Parameter tuning was performed comparing the performance of ANN with a different number of both hidden layers (from 0 to 1) and neurons (2 to 6). Moreover, the learning rate was let to vary between 0.25 and 0.75 (step 0.05).

#### K-nearest neighbor classifier (kNN)

Let *n* be the number of pairs (***x***_*j*_,*y*_*j*_) in the training set *S*, where ***x***_*j*_ is the input vector and *y*_*j*_ the output class for the *j*th sample. When a new subject described by the input vector ***x*** is to be classified, the nearest *k* samples in *S*, according to a suitable distance measure, are determined and the class *y* associated with the majority of the *k* nearest samples is assigned to ***x*** [32].

In the present investigation the standard Euclidean distance was employed, after having normalized the components of the input vector ***x*** to reduce the effect of biases possibly caused by unbalanced domain intervals for different input variables. Tuning procedure was applied to the number of nearest samples letting the *k* parameter vary between 1 to 10.

#### Support vector machine (SVM)

SVM is a non-probabilistic binary linear classifier based on the identification of an optimal hyperplane of separation between two classes [32]. Given a training set, the classifier selects a subset *l* of input vectors ***x***_*j*_ in the training set *S*, called support vectors, and their corresponding outputs *y*_*j*_ ∈ {− 1,1}. The class *y* for any input vector ***x*** is then given by:

$$ y=\operatorname{sgn}\left(\sum \limits_{j=1}^l{y}_j{\alpha}_jK\left({\boldsymbol{x}}_j,\boldsymbol{x}\right)+b\right) $$

where the coefficients *α*_*j*_ and the offset *b* are evaluated through a proper training algorithm.

*K*(*·*,*·*) is a kernel function used to perform a non-linear classification by constructing an optimal hyperplane in a high dimensional projected space. A linear kernel function was tested on each dataset. The training algorithm was performed by using the LIBSVM library, which is featured by the Rulex Analytics software. The performance of SVM with linear and RBF kernels was tested. Tuning procedure also included the degree of the kernel function that was let range from 1 to 10.

## Data Availability

The datasets analyzed during the current study are available in the GEO repository bank: https://www.ncbi.nlm.nih.gov/sites/GDSbrowser?acc=GDS4968 https://www.ncbi.nlm.nih.gov/sites/GDSbrowser?acc=GDS4887 https://www.ncbi.nlm.nih.gov/sites/GDSbrowser?acc=GDS4794 https://www.ncbi.nlm.nih.gov/sites/GDSbrowser?acc=GDS4762 https://www.ncbi.nlm.nih.gov/sites/GDSbrowser?acc=GDS4471 https://www.ncbi.nlm.nih.gov/sites/GDSbrowser?acc=GDS4296 https://www.ncbi.nlm.nih.gov/sites/GDSbrowser?acc=GDS3952 https://www.ncbi.nlm.nih.gov/sites/GDSbrowser?acc=GDS3945

## Abbreviations

ANN: Artificial Neural Network
DT: Decision Tree
KNN: k-Nearest Neighbor classifier
LLM: Logic Learning Machine
LOOCV: Leave-One-Out Cross Validation
sAUC: Summary Area Under the ROC Curve
sROC: Summary Receiver Operating Characteristic curve
SVM: Support Vector Machine
